# Hematology and Plasma Chemistry Reference Values in Nursehound Shark (*Scyliorhinus Stellaris*) Maintained Under Human Care

**DOI:** 10.3389/fvets.2022.909834

**Published:** 2022-07-11

**Authors:** Pablo Morón-Elorza, Carlos Rojo-Solis, Christine Steyrer, Teresa Álvaro-Álvarez, Mónica Valls-Torres, Teresa Encinas, Daniel García-Párraga

**Affiliations:** ^1^Department of Pharmacology and Toxicology, Faculty of Veterinary Medicine, Complutense University of Madrid, Madrid, Spain; ^2^Fundación Oceanogràfic de la Comunitat Valenciana, Valencia, Spain; ^3^Veterinary Services, Oceanogràfic, Ciudad de las Artes y las Ciencias, Valencia, Spain; ^4^Lionsrock Big Cat Sanctuary, Bethlehem, South Africa

**Keywords:** elasmobranch, shark, hematology, biochemistry, leukocyte, granulocyte, Abaxis, Zoetis

## Abstract

Studies determining baseline hematological reference intervals (RI) in elasmobranchs are very limited. In this study, blood samples were collected from 94 clinically healthy Nursehound Shark (*Scyliorhinus stellaris*) maintained under human care. Median (RI) in major leukocyte types were similar to other Carcharhinid sharks as lymphocytes were the predominant leukocyte with 38.0 (28.2–53.5)%, followed by coarse eosinophilic granulocytes with 20.0 (12.2–31.7)%, fine eosinophilic granulocytes with 6.0 (1.2–12.8) %, monocytes with 2.0 (0.0–6.0)%, and neutrophils with 2.0 (0.0–6.0)%. Nursehound Shark produced granulated thrombocytes, which were classified as granulocytes and represented 28.5 (12.4–39.7)% of all leukocytes. Median (RI) manual red blood cell and white blood cell counts were 177.50 (132.50–210.00) x 10^9^ cells/l and 8.26 (5.24–14.23) x 10^9^ cells/l, respectively. Median (RI) plasma chemistry values showed alkaline phosphatase 7.7 (4.2–13.0) U/l, aspartate aminotransferase 7.6 (3.3–17.1) U/l, blood urea nitrogen 281.6 (261.2–305.0) mmol/l, calcium 3.97 (3.59–4.47) mmol/l, total cholesterol 2.04 (1.02–3.91) mmol/l, chloride 233.0 (215.2–259.0) mmol/l, iron 3.79 (1.74–6.93) μmol/l, glucose 0.87 (0.47–1.44 mmol/l), potassium 3.8 (2.9–4.6) mmol/l, sodium 243.0 (227.7–271.0) mmol/l, phosphorus 1.58 (1.13–2.10) mmol/l, total protein 24.0 (20.0–35.0) g/l, and triglycerides 0.97 (0.49–3.35) mmol/l. Creatine kinase, gamma glutamyl transferase, and lactate dehydrogenase levels were below the instrument reading range.

## Introduction

Population-based reference values (RV) represent the most widely used tool for the interpretation of individual clinical laboratory test results and are typically reported as reference intervals (RI) comprising 90–95% of a healthy reference population (1, 2). After the introduction of the concept in human medicine in 1969, RV were rapidly determined for humans and most companion animal species (3–6). However, accurate RV are still lacking for most exotic and aquatic animal species (7). Despite the universally accepted use of population-based RV, the optimal method for their determination is often discussed, and although a reference population of 120 individuals is frequently recommended for the establishment of species-specific RV, the use of a smaller number of reference individuals is accepted as it is often very difficult to reach large numbers of specimens in many non-domestic animal species (1, 8). While the accuracy in the estimation of RI decreases with the reduction in the reference population size, RI can be statistically determined using parametric or robust statistical methods with sample sizes as low as 20–40 reference individuals (1, 9). Provided that a sample size of 40–120 individuals can be achieved, parametric, robust, or non-parametric methods can be used for the RI determination (1).

Blood collection should be included in the routine health evaluation of elasmobranchs, as underlying disease diagnosis in fish can be challenging and blood work adds important information for their precise health status determination (8, 10, 11). For this purpose, the determination of blood RV provides an important tool for veterinarians, biologists and professionals working with these animals (10). Yet, due to the challenges of working with elasmobranchs, and the difficulties in having access to a large and homogeneous population of sharks and rays, most studies describing hematological and plasma biochemical RI in these animals often rely on small study populations (frequently under 30 individuals) (12–16). In addition, significant differences have been reported among studied species, elucidating the need to obtain species-specific RI to guarantee accurate health assessment interpretations in these animals and the need for further studies with larger elasmobranch populations (15). Collaborations between aquaria maintaining the same elasmobranch species in similar environmental conditions facilitate the obtention of larger reference sample sizes and can allow the determination of more reliable RV.

The Nursehound Shark (*Scyliorhinus stellaris*) of the order *Carcharhiniformes* is a medium-sized oviparous catshark found in the Northeast Atlantic Ocean and the Mediterranean Sea (17). It is classified as Vulnerable by the IUCN Red List (last consulted in March 2022), with a decreasing population trend (18). Although the species has been consistently housed in aquaria since the 1860s, studies involving this species are still quite limited in comparison to the frequently studied Smallspotted Catshark (*Scyliorhinus canicula*) (19). In addition, to the authors' knowledge, population-based RV have not been determined for this species.

The objective of this study was to document the main hematological and plasma chemical RV for the Nursehound Shark, as well as possible differences which could be associated with sex and developmental stage, using a population of healthy individuals maintained under human care. This information will allow a more accurate interpretation of blood analytical values and improve the quality of the health evaluations performed in this species. Additionally, it will provide a better understanding and comparison of hematology and plasma chemistry with other studied elasmobranch species. It will also allow for further comparison to *in situ* studies with free-ranging Nursehound Shark.

## Materials and Methods

All procedures were approved by the Animal Care and Welfare Committee at Oceanogràfic of Valencia and the Generalitat Valenciana under the project reference OCE-22-19.

### Study Design

This study was designed as a prospective experimental trial, arranged as a collaboration between five different Spanish aquaria which housed Nursehound Shark: Oceanogràfic Aquarium of Valencia (Carrer d'Eduardo Primo Yúfera, 1B, Valencia 46013, Spain; www.oceanografic.org), Finisterrae Aquarium (P.° Marítimo Alcalde Francisco Vázquez, 34, A Coruña 15002, Spain; www.coruna.gal/mc2/es/aquarium-finisterrae), Bioparc Gijón Aquarium (Playa De Poniente, S/N, Gijón 33212, Spain; www.acuariogijon.es); Museo Marítimo del Cantábrico (Av. de Severiano Ballesteros, s/n, Santander 39004, Spain; www.museosdecantabria.es/museo-maritimo), and Sevilla Aquarium (Muelle de las Delicias, s/n, Sevilla 41012, Spain; www.acuariosevilla.es).

### Animals, Sample Collection and Processing

We collected blood samples from 94 clinically healthy Nursehound Shark (47 females and 47 males). We determined gender based on the presence of claspers (copulatory organs only present in males) (20). We determined body weight (kg) using a crane scale (GRAM CR 150-S, Gram Precision S.L., l'Hospitalet de Llobregat 08907, Spain), and we took measures for total length (snout to tip of the tail; TL, in cm), and snout to vent length (snout to cloaca; SVL, in cm) using a flexible nylon measure. Former studies determined that female Nursehound Shark mature at an approximate total length of 79 cm, while males mature at 77 cm (17). Total length in animals sampled in our study ranged from 51.0 to 113.0 cm. According to this, we classified sampled shark into two life-history stages: adult (*n* = 54; 25 females and 29 males) (females with a TL over 79 cm and males with a TL over 77 cm) or subadult (*n* = 40; 22 females and 18 males) (females with a TL under 79 cm and males with a TL under 77 cm), to examine possible differences in their analytical values. It was not possible to accurately classify animals as sexually mature/immature nor determine if adult females were carrying eggs. Sampled animals were housed in five different aquaria in Spain. Table 1 provides demographic information for sampled shark.

**Table 1:** Classification of Nursehound Shark (*Scyliorhinus stellaris*) included in this study according to sex and developmental stage.

**Aquarium**	**Sex**		**Developmental stage**	
	**Female**	**Male**	**Adult**	**Subadult**
Oceanogràfic Aquarium	6	10	14	2
Finisterrae Aquarium	15	7	14	8
Bioparc Gijón Aquarium	5	5	5	5
Mueo Marítimo del Cantábrico	10	7	16	1
Sevilla Aquarium	11	18	5	24
Total	47	47	54	40

*Shark were sampled from five different aquaria in Spain. Sex was determined based on the presence of claspers and developmental stage was determined based on their total length (17, 20)*.

We selected reference individuals after the evaluation of their medical history and the completion of an initial physical examination to assess their general condition. Any deviation in an animal's medical history, behavior, skin color and appearance, ocular status and reflexes, oral cavity, gums and teeth appearance, presence of external lesions, spinal deformities, abnormal swimming, or buccal pumping/forced ventilation was considered an exclusion criteria. All shark were considered clinically healthy, and the 94 collected samples were used for determining the RI. All sampling occurred during the month of August 2021. We submitted all individuals to a fasting period which ranged from 24 to 36 h prior to sampling, as imbalances in plasma chemistry have been described in the Spiny dogfish (*Squalus acanthias*) when samples were collected after feeding (21).

Environmental conditions varied slightly between aquaria included in the study. In all aquaria, shark were maintained in tanks provided with life support systems which included mechanical filtration (through sand filters), protein skimmers, biological filtration, and disinfection through UV. In all aquaria, ammonia was maintained under 0.01 ppm, nitrite under 0.05 ppm, and nitrate under 100 ppm. Holding systems in Finisterrae Aquarium, Bioparc Gijón Aquarium, and Museo Marítimo del Cantábrico were filled with natural sea water, filtered from the Cantabrian Sea (coastal waters of the Atlantic Ocean), while Oceanogràfic Aquarium was supplied with natural seawater filtered from the coast of Valencia (Mediterranean Sea) and Sevilla Aquarium was supplied with water filtered from the coast of Cadiz, Spain (Atlantic Ocean). Water temperature, pH, and salinity in the holding systems of Oceanogràfic Aquarium were 18°C, 8.0 and 35.0 ‰, respectively; 18.5°C, 7.9, and 35.2 ‰ in Finisterrae Aquarium; 13°C, 8.0, and 35.0 ‰ in Bioparc Gijón Aquarium; 19°C, 7.8 and 35.6 ‰ in Museo Marítimo del Cantábrico; 18°C, 8.1 and 34.5 ‰ in Sevilla Aquarium. Diet also varied slightly between the different aquaria: Nursehound Shark housed at Oceanogràfic and Sevilla Aquariums were fed once daily, 3 days per week, with pieces of thawed hake (*Merluccius spp*.), squid (*Loligo spp*.), herring (*Clupea spp*.), or mackerel (*Scomber spp*.) supplemented with potassium iodide capsules and Akwavit® fish eating animal tablets (Product reference: 690,206; KASPER faunafood, Woerden 3,440 AA, Netherlands). Nursehound Shark housed at Finisterrae Aquarium were fed once daily, 3 days per week, with pieces of thawed hake, mackerel, and prawn (*Penaus spp*.) supplemented with Vetregard® immunostimulants (AQUAVET S.A., Athens 14,341, Greece), Vit-amin C and Mazuri® Shark & Ray tablets (Mazuri® exotic animal nutrition; St. Louis 63,166, USA). Shark housed at Bioparc Gijón Aquarium were fed once daily, 3 days per week, with pieces of thawed hake, squid, capelin (*Mallotus spp*.), and sprat (*Sprattus sp*p.); feed trays were supplemented with spirulina and Calfostonic® powder (Industrial Veterinaria, S.A, Esplugues de Llobregat 08950, Spain). Finally, shark housed at Museo Marítimo del Cantábrico were fed once daily, 4 days per week with pieces of thawed mackerel, Humboldt squid (*Dosidicus gigas*), and pollock (*Pollachius spp*.). All aquaria participating in the study maintained a similar feeding intake rate for their Nursehound Shark, ranging from 5 to 10% body weight per week.

During sampling, we carefully captured shark using a rubber net and manually restrained them in water in a dorsal recumbency to induce tonic immobility which occurred in <1 min in all sampled individuals facilitating blood collection (22). We collected a volume of 1 ml peripheral blood immediately from the caudal vasculature by lateral approach using a 25-gauge needle attached to a 1 ml syringe (22, 23). Blood was then introduced into 1 ml lithium heparin tubes (AQUISEL® 1 ml 12x55mm REF: 1,501,406; Aquisel S.L., Abrera 08630, Spain), which were refrigerated at 4°C and processed within a maximum of 90 min after collection. Time required from capture until blood collection was always <3 min (ranging from 1 to 3 min), and the entire handling procedure was always <5 min per individual (ranging from 3 to 5 min). No animal showed clinical signs during the study nor the month following the study.

Hematological analysis included manual packed cell volume (PCV), which we determined using microhematocrit tubes, centrifuged at 1,372 g (3,500 rpm) for 6 min at room temperature (24°C) in a portable centrifuge (Digital angle centrifuge 2,615/1 Nahita-Blue, 100 mm rotor radius; LABOQUIMIA, Lardero 26,140, Spain). We determined plasma total solids (TS) using an analog refractometer (LABOLAN® model FG301/311; Labolan S.L., Esparza 31,191, Spain). Next, we collected 10 μl of blood and mixed with them with 490 microliter Natt-Herricks solution (Natt-Pette™, Exotic Animal Solutions, Inc., Melbourne 32,941, USA) to perform RBC and total granulocyte manual counts using a Neubauer improved counting chamber (Assistant Glaswarenfabrik Karl Hecht Gmbh & Co Kg, Sondheim vor der Rhön 97,647, Germany). We performed blood smears using lithium-heparin blood samples, previously homogenized, left to dry at ambient temperature (24°C), and stained using a Diff-Quick stain (Diffvet DMV Diagnóstico Médico Veterinario, SL., Barcelona 08198, Spain). We also performed leukocyte differential counts using a portable optical microscope (Vetscan HDmicroscope, ABAXIS Europe Gmbh, Griesheim 64,347, Germany) equipped with an IS E-Plan achromat objective 100 x and immersion oil. We inspected each smear to exclude the presence of platelet and leukocyte aggregates. Leukocyte differentials consisted of counting a minimum of 100 WBC per animal and sample, and the further calculation of cell percentages. Scyliorhinid shark leukocytes have been studied under electron microscopy in previous studies and have been classified into four types according to the structure of their granules (24). In this study, we performed leukocyte differential counts using the following cell nomenclature: fine eosinophilic granulocyte (FEG, granulocyte type I or heterophil-like cells); neutrophil (granulocyte type II or neutrophil-like cells), coarse eosinophilic granulocyte (CEG, granulocyte type III or eosinophil-like cells), granulated thrombocytes (GT, granulocyte type IV), lymphocytes, and monocytes (13, 24).

After hematology analysis, we centrifuged lithium heparin tubes at 448 g (2,000 rpm) for 5 min to obtain plasma using the same centrifuge. We collected plasma, transferred it to 1.5 ml Eppendorf tubes, which were frozen at −18°C until processing. We Processed all samples within a maximum of 30 days from collection at the laboratory located at the veterinary clinic of Oceanogràfic Aquarium using a Beckman Coulter® Chemistry Analyzer through spectrophotometry and potentiometry (A91961 - AU480 Chemistry Analyzer, Beckman Coulter S.L.U., Alcobendas 28,108, Spain). Plasma chemistry profiles included alkaline phosphatase (ALP) (International Federation of Clinical Chemistry -IFCC- method), aspartate aminotransferase (AST) (IFCC method with Pyridoxal Phosphate), blood urea nitrogen (BUN) (Urease/GLDH method), calcium (Ca) (oCPC method), total cholesterol (CHO-POD method), chloride (Cl) (ISE indirect), creatine kinase (CK) (IFCC method), gamma-glutamyl transferase (GGT) (IFCC method), glucose (Gluc) (HK G6P-DH method), iron (Fe) (TPTZ method), lactate dehydrogenase (LDH) (IFCC method), sodium (Na) (ISE indirect), phosphorus (molybdate, UV method), potassium (K) (ISE indirect), total proteins (TP) (Biuret method), and triglycerides (Trig) (GPO-POD method).

### Statistical Analysis

We established reference intervals following the recommended guidelines published by the American Society of Veterinary Clinical Pathology (ASCVP), using the RefVal adv. 2.0 Excel addin and the MedCalc® statistical software (1, 9). We identified outliers using the Dixon's outlier range statistic, and we performed a visual inspection of histograms (25). If we detected an outlier in one analyte, iwas eliminated it and re-run the analysis until the dataset was free from outliers. For leukocyte differential counts, if one outlier was detected in one cell type, we excluded the entire differential count from that individual from the leukocyte differential count. We used the Shapiro-Wilk test to determine Gaussian distribution and established a *p*-value of 0.2 as a cut off value instead of 0.05, following the previously established recommendations for RI determination using small sample sizes (26). If *p* ≥ 0.2, the parametric method was used, and if *p* ≤ 0.2 the non-parametric method was used. The 90% confidence intervals (CI) were calculated using a bootstrap method. Because all obtained data were not normally distributed, we used nonparametric statistics, and we report summary statistics as median values throughout the manuscript. We also provide mean values, SD, maximum, and minimum values for every analyte in the tables presented in the results section of this manuscript. We evaluated differences between sexes (female vs. male) and developmental stage (adult vs. subadult) for morphometrical, hematological, and biochemical analytes using a Mann-Whitney U test. We evaluated differences between aquaria and subgroups (adult female vs. adult male vs. subadult female vs. subadult male) using a Kruskal-Wallis test and Dunn's *post hoc* tests with Bonferroni corrections. In addition, we performed a Spearman's rank-order correlation, and calculated the coefficient of determination (R^2^) for size (using the morphometric measurements weight, TL and SVL) and the rest of the hematological and plasma chemical parameters. We performed statistical analyses using the statistical software package RStudio® (Version 1.2.504; RStudio Team, 2020. Boston 02210, USA; www.rstudio.com). We set statistical significance at *p* < 0.05. We have expressed results using the Standard International System of Units.

## Results

### Morphometric Measurements

Median weight was 2.40 kg (range = 0.70–7.45 kg), median TL was 79.0 cm (range = 51.0–113.0 cm), median SVL was 38.0 cm (range = 18.0–56.0 cm). A comparison for morphological measurements between female and male sharks is provided in the Supplementary Material. No statistically significant differences were detected between sexes for weight, TL, and SVL (*p* = 0.817, 0.607, and 0.522, respectively; Mann-Whitney *U* test). When comparing across subgroups (adult females vs. adult males vs. subadult females vs. subadult males), differences were also not significant (*p* > 0.05; Dunn's test, Bonferroni adjusted).

### Hematology

**Figure 1:**
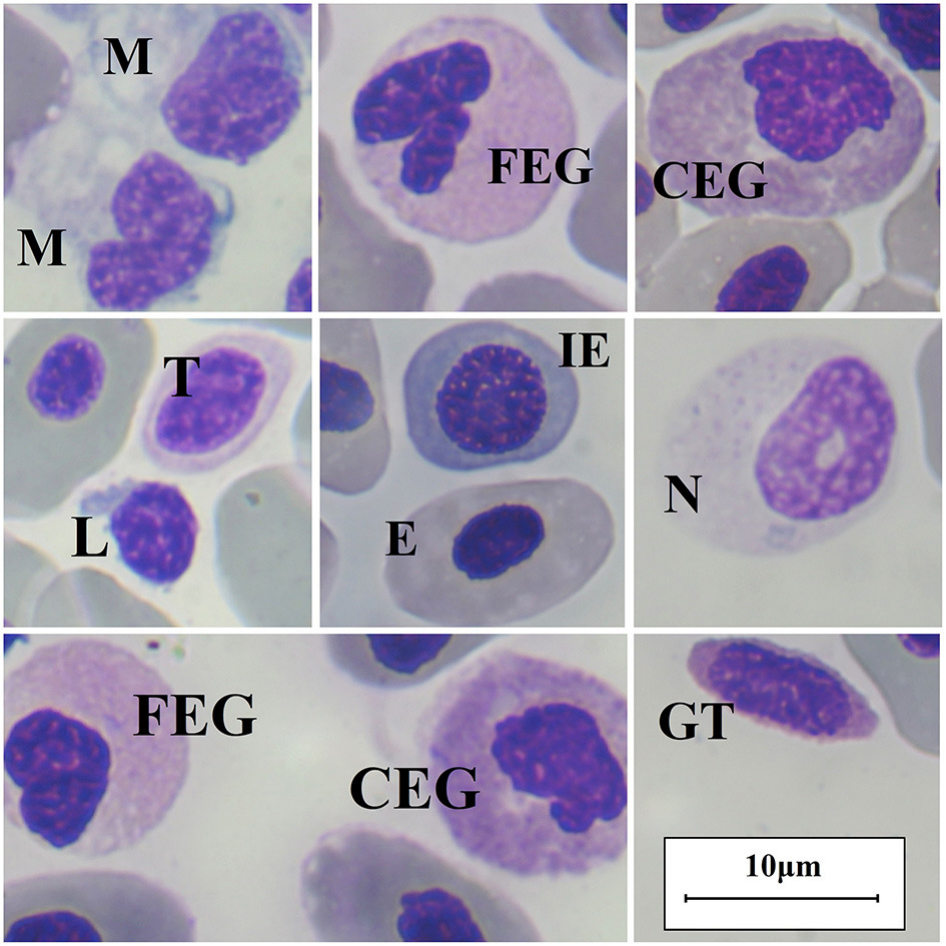
Nursehound Shark (*Scyliorinus stellaris*) main peripheral blood cells. Diff-Quick stain and optic microscopy 100x using immersion oil. M, monocyte; FEG, fine-eosinophilic granulocyte; CEG, coarse-eosinophilic granulocyte; L, lymphocyte; T, thrombocyte; E, erythrocyte (mature); IE, immature erythrocyte; N, neutrophil; GT, granulated thrombocyte.

Descriptive statistics for hematology analytes are shown in Table 2, a comparison for hematological values between sexes and a comparison between developmental stages is provided in the Supplementary Material. We classified leukocytes as lymphocytes, monocytes, neutrophils, FEG and CEG. Nursehounds produced granulated thrombocytes, which were classified as granulocytes (13, 24). We did not find statistically significant differences between sexes for any hematological value (*p* > 0.05; Mann-Whitney *U* test), and we did not detect significant differences when comparing the hematological values obtained from the different aquaria participating in the study (*p* > 0.05; Kruskal-Wallis test). Furthermore, we did not detectstatistically significant differences in hematological parameters between developmental stages (adult vs. subadult) (*p* > 0.05; Mann-Whitney *U* test) and across the different subgroups (adult female vs. adult male vs. subadult female vs. subadult male) (*p* > 0.05; Kruskal-Wallis test). Main peripheral blood cells found in the Nursehound Shark are presented in Figure 1.

**Table 2:** Hematology reference intervals for Nursehound Shark (*Scyliorhinus stellaris*) under human care.

**Analyte (unit)**	***n***	**Mean**	**SD**	**Median**	**Min**	**Max**	**RI**	**LRL**	**URL**
PCV (l/l)	94	0.17	0.01	0.17	0.14	0.21	0.15–0.20	0.14–0.15	0.20–0.21
TS (g/l)	94	58.0	4.0	58.0	50.0	68.0	50.0–66.0	50.0–51.0	66.0–68.0
WBC (10^9^/l)	86	8.85	2.53	8.26	4.91	14.53	5.21–14.23	4.91–5.66	13.47–14.53
RBC (10^9^/l)	94	172.7	22.4	177.5	122.5	240.0	132.5–210.0	122.5–135.9	205.0–240.0
L (%)	86	39.5	5.5	38.0	27.0	54.0	28.2–53.5	27.0–32.0	48.0–54.0
M (%)	86	2.8	1.6	2.0	0.0	7.0	0.0–6.0	0.0–0.2	6.0–7.0
N (%)	86	2.5	1.6	2.0	0.0	6.0	0.0–6.0	0.0–0.0	5.7–6.0
FEG (%)	86	6.3	2.8	6.0	1.0	14.0	1.2–12.8	1.0–3.0	12.0–14.0
CEG (%)	86	20.7	4.6	20.0	12.0	33.0	12.2–31.7	12.0–14.0	28.0–33.0
GT (%)	86	28.2	6.4	28.5	12.0	40.0	12.4–39.7	12.0–16.5	38.0–40.0
L (10^9^/l)	84	3.44	1.15	3.24	1.55	6.55	1.80–6.48	1.55–1.97	5.68–6.55
M (10^9^/l)	86	0.25	0.15	0.23	0.00	0.65	0.00–0.61	0.00–0.01	0.51–0.65
N (10^9^/l)	84	0.21	0.13	0.18	0.00	0.56	0.00–0.52	0.00–0.00	0.41–0.56
FEG (10^9^/l)	86	0.55	0.27	0.48	0.09	1.32	0.12–1.28	0.09–0.22	1.18–1.32
CEG (10^9^/l)	86	1.82	0.63	1.71	0.68	3.67	0.81–3.60	0.69–1.09	2.93–3.68
GT (10^9^/l)	85	2.46	0.84	2.33	0.75	4.50	1.12–4.37	0.75–1.28	3.99–4.50

*n, number of individuals; SD, standard deviation; RI, reference intervals; LRL, 90% confidence interval of the lower reference limit; URL, 90% confidence interval of the upper reference limit; D, distribution; PCV, packed cell volume; TS, total solids; WBC, white blood cells; RBC, red blood cells; L, lymphocyte; M, monocyte; FEG, fine eosinophilic granulocyte; CEG, coarse eosinophilic granulocyte; N, neutrophile; GT, granulated thrombocyte*.

### Blood Chemistry

Descriptive statistics for blood chemistry are presented in Table 3, and a comparison between female and male shark biochemical values is presented in the Supplementary Material. Plasma CK and LDH values are not reported as they were under the limit of detection in all sampled animals (10 U/l for CK and 25 U/l in LDH). Plasma GGT values are reported in this study, despite being under the linear ranges of the chemistry analyzer (3–1,200 U/l), as they were consistent in all animals sampled and maintained within values previously determined in several elasmobranch species (27). Median plasma total cholesterol was significantly higher in male shark (2.17 mmol/l) compared to females (1.71 mmol/l) (*p* < 0.05; Mann-Whitney U test). Plasma total cholesterol was also significantly higher in adult males (median 2.36 mmol/l) when compared to adult females (median 1.57 mmol/l) (*p* < 0.05; Dunn's test, Bonferroni-adjusted), but comparison between subadult males (median 2.12 mmol/l) and subadult females (median 1.86 mmol/l) showed no significant differences (*p* = 0.295; Dunn's test, Bonferroni-adjusted), thus revealing that differences in cholesterol between sexes were stronger in adult animals. No statistically significant differences were detected when comparing total cholesterol levels between adult males vs. subadult males (*p* = 0.850; Dunn's test, Bonferroni-adjusted) nor between adult females vs. subadult females (*p* = 0.120; Dunn's test, Bonferroni-adjusted). Except total cholesterol, no statistically significant differences in any other parameter were detected between sexes. Variations in plasma chemical values between aquaria were only statistically significant for ALP (*p* < 0.05; Dunn's test, Bonferroni-adjusted). Correlation between size (using the determined morphometric measurements: weight, TL, and SVL) and biochemical parameters was low (R^2^ < 0.150). Despite this low correlation, statistically significant differences were found for GGT between adult and subadult shark (*p* < 0.05; Mann-Whitney *U* test).

**Table 3:** Blood chemistry reference intervals for Nursehound Sharks (*Scyliorhinus stellaris*) under human care.

**Analyte (unit)**	***n***	**Mean**	**SD**	**Median**	**Min**	**Max**	**RI**	**LRL**	**URL**
ALP (U/l)	92	7.8	2.2	7.7	4.1	13.8	4.2–13.0	4.1–4.6	11.7–13.8
AST (U/l)	88	8.0	3.5	7.6	3.0	17.2	3.3–17.1	3.0–3.5	15.3–17.2
BUN (mmol/l)	94	281.9	12.5	281.6	256.0	315.7	261.2–305.0	256.0–265.0	302.4–315.7
Ca (mmol/l)	89	3.97	0.20	3.97	3.52	4.62	3.59–4.47	3.52–3.64	4.37–4.62
Chol (mmol/l)	93	2.07	0.74	2.04	0.60	4.04	1.02–3.91	0.60–1.10	3.68–4.04
Cl (mmol/l)	86	233.5	10.0	233.0	213.0	259.0	215.2–259.0	213.0–218.4	254.0–259.0
Fe (μmol/l)	94	3.79	1.34	3.79	1.27	7.00	1.74–6.93	1.27–2.17	6.48–7.0
GGT (U/l)	91	0.9	0.8	0.6	0.0	2.8	0.0–2.6	0.0–0.1	2.5–2.8
Gluc (mmol/l)	90	0.89	0.23	0.87	0.23	1.44	0.47–1.44	0.44–0.52	1.34–1.44
K (mmol/l)	94	3.8	0.4	3.8	2.8	4.8	2.9–4.6	2.8–3.1	4.4–4.8
Na (mmol/l)	92	244.7	11.1	243.0	224.0	272.0	227.7–271.0	224.0–230.0	269.0–272.0
Phos (mmol/l)	94	1.58	0.26	1.58	1.00	2.13	1.13–2.10	1.00–1.19	2.03–2.13
TP (g/l)	94	25.0	4.0	24.0	19.0	36.0	20.0–35.0	19.0–20.0	33.0–36.0
Trig (mmol/l)	90	1.17	0.65	0.97	0.39	3.44	0.49–3.35	0.39–0.55	2.51–3.44

*n, number of individuals; SD, standard deviation; RI, reference intervals; LRL, 90% confidence interval of the lower reference limit; URL, 90% confidence interval of the upper reference limit; ALP, alkaline phosphatase; AST, aspartate aminotransferase; BUN, blood urea nitrogen; Ca, calcium; Chol, total cholesterol; Cl, chloride; GGT, gamma-glutamyl transferase; Gluc, glucose; Fe, iron; Na, sodium; Phos, phosphorus; K, potassium; TP, total proteins; Trig, triglycerides. Reported GGT values were under the linear range of detection of the chemistry analyzer (3-1200 U/l)*.

## Discussion

Aquaria allow for the achievement of safe, efficient, and economic study of elasmobranchs, as well as procurement of important information and expansion of knowledge in many areas of elasmobranch biology and medicine (11, 28).

### Hematology

Median PCV (0.17 l/l) values were within ranges of those reported in previous studies with elasmobranchs, which show typically low values in healthy elasmobranchs and important variations between species (aquarium-maintained Bamboo Shark (*Chiloscyllium plagiosum*) (0.13 l/l), aquarium-maintained Smooth Dogfish (*Mustelus canis*) (0.24 l/l), and free-ranging Bonnethead Shark (*Sphyrna tiburo*) (0.22 l/l) (16, 29, 30). Median TS (58 g/l) were also similar to other elasmobranch species such as the free-ranging Bonnethead Shark (63 g/l), the free-ranging Southern Stingray (*Dasyatis americana*) (56 g/l) and the aquarium-maintained Spotted Eagle Ray (*Aetobatus narinari*) (57 g/l) (8, 16, 31). Variations in elasmobranch RBC counts across species have been associated with differences in physiological activity (32). Median RBC counts (177.50 x 10^9^ erythrocytes/l) were similar to those obtained in other non-obligate ram-ventilating benthic elasmobranchs such as the Winter Skate (*Leucoraja ocellata*) (200.0 x 10^9^ erythrocytes/l), and lower than those reported in pelagic elasmobranch species such as the Cownose Ray (*Rhinoptera bonasus*) (511.2 x 10^9^ erythrocytes/l) and other obligate ram-ventilating Carcharhinid sharks such as the Sandbar Shark (*Carcharhinus plumbeus*) (402.0 x 10^9^ erythrocytes/l) and the Blacktip Shark (*Carcharhinus limbatus*) (476.0 x 10^9^ erythrocytes/l) (10, 32–34). Capture-related alterations in PCV and RBC counts should be also considered in elasmobranchs, as an increase in PCV and RBC has been reported in different shark species during exercise and after exposure to anoxic conditions, which have been associated to RBC release from hematopoietic organs into the peripheral blood (35).

Former hematological studies with elasmobranchs also showed important variations in leucocyte differential counts between the different elasmobranch species (10, 15). Lymphocytes, monocytes, FEG, and CEG appear to be present in all the studied elasmobranch species, though their proportions in the leukocyte differential counts vary greatly across shark and ray species (13, 36, 37). In our study, median proportion of the major leukocyte types was similar to other Carcharhinid sharks, with lymphocytes as the predominant WBC with 38.0% of all leukocytes, followed by CEG with 20.0%, FEG with 6.0%, monocytes with 2.0%, and neutrophils with 2.0%. As previously described in other Carcharhinid species, the Nursehound Shark had granulated thrombocytes, which in an attempt to assist the standardization of hematological analysis in elasmobranchs, were classified as granulocytes and represented 28.5% of all leukocytes (30). Granulated thrombocytes have also been reported as the most frequent granulocyte in other Carcharhinid sharks such as the aquarium-maintained Sandbar Shark, with a median percentage of 21% in the differential count, followed by CEG (11.0%), FEG (7.0%), and neutrophils (6.5%); lymphocytes were also the predominant peripheral leukocyte (50.0%) and with monocyte production occurring only in small pro-portions (3.0%) (13). Previous studies also described 2 types of thrombocytes in other elasmobranchs, including the Smallspotted Catsharks, the Torpedo Ray (*Torpedo spp.)*, and the Port Jackson Shark (*Heterodontus portusjacksoni*) (24, 38, 39). However, GT seem to be absent in other Non-Carcharhinid elasmobranch species such as the aquarium-maintained Cownose Ray and the aquarium-maintained Whale Shark (*Rhincodon typus*), where FEG have been reported as the most frequent peripheral granulocyte (36, 37). Granulated thrombocytes do not have a clear mammalian equivalent cell and their specific role in elasmobranchs remains unknown (30, 35). Similar to Nursehound Shark, some other species such as the aquarium-maintained Smooth Dogfish, the Sandbar Shark, and the Whale Shark also produce a granulocyte type, classified as neutrophil, in which the granules do not take up stain (13, 37). Basophils were not detected in Nursehound Shark peripheral blood smears. Basophils, described as extremely rare or absent in some elasmobranch species, are also not included in the differential counts of Carcharhinid sharks such as the Sandbar Shark and the aquarium-maintained Smooth Dogfish (10, 13, 30). Interpreting leukocyte differential counts in elasmobranchs in challenging, as the clinical implications of the leukocyte types is not fully understood. Leukocytosis has been reported in elasmobranchs suffering from stress and inflammation related to trauma, bacterial, fungal or parasitic infections (35). Monocytes and FEG obtained from the Nurse Shark (*Ginglymostoma cirratum*) have demonstrated phagocytic activity *in vitro*, and despite immature granulocytes are described as a normal finding in blood smears from clinically healthy elasmobranchs, a left-shift in leukocyte differential counts has been also described in elasmobranchs suffering from infectious and inflammatory pathologies (35, 40).

Total leukocyte counts (median 8.26 x 109 cells/l) were kept within the previously reported values for other elasmobranch species such as the aquarium-maintained Whale Shark (5.1 x 109 cells/l) and the Blacktip Reef Shark (*Carcharhinus melanopterus*) (13.7 x109 cells/l) (15, 39, 41). As a reference, blood samples collected from injured female Nursehound Shark with moderate reproductive bite wounds at Oceanogràfic Aquarium, which were not included in this study, provided examples of data outside the determined RI for the species. For instance, an adult Nursehound Shark (2.7 kg, 84 cm TL and 40 cm SL) had increased WBC counts (21.34 x 109 cells/l) and FEG percentage (23%). WBC counts and FEG percentages have been described to increase in fish suffering from infectious or inflammatory diseases and stress (42, 43). However, these increased values for the studied species would still be considered within range if hematological values were extrapolated from median WBC counts (min-max) previously determined for the aquarium-maintained Smooth Dogfish (15.3 (10.5–23.9) x 109 cells/l), the Sandbar Shark (22.1 (17.2–26.7) x 109 cells/l), the Grey Nurse Shark (*Carcharias taurus*) and the Tiger shark (*Galeocerdo cuvier*) (16.3 (4.5–40.5) x 109 cells/l) (33, 44). The large differences detected in hematological values across elasmobranch species, including leukocyte differential counts, RBC counts, and WBC counts, illuminate the limitations of inter-specific extrapolation when working with sharks and rays and the need for further studies determining hematological RV in a wide range of elasmobranch species.

### Blood Chemistry

Statistically significant variations in plasma chemical values were only detected across aquaria for ALP, with significantly higher ALP in animals sampled from Bioparc Gijón Aquarium (median 10.9 U/l) compared to Museo Marítimo del Cantábrico (5.6 U/l) and Finisterrae Aquarium (median 7.3 U/l) (*p* < 0.05, Dunn's test, Bonferroni-adjusted). Variations were not significant when compared to Oceanogràfic of Valencia (median 7.8 U/l) and Sevilla Aquarium (median 7.9 U/l). Alkaline phosphatase activity is involved in the mineralization of both bony and cartilaginous skeletons (27, 45). Different factors could have contributed to the observed differences in ALP values as variations related to diet, growth, and skeletal development have been reported in elasmobranchs (12, 27). Bioparc Gijón Aquarium was the only aquarium supplementing their animals with Calfostonic® powder (CALFOSTONIC, Industrial Veterinaria, S.A, 08950 Esplugues de Llobregat, Spain) which is rich in calcium, phosphorus, and vitamin D3 (cholecalciferol), among other analytical components. Alkaline phosphatase activity has proven to increase 2–3-folds in chicks after the administration of vitamin D3, and interactions between vitamin D and calcium metabolism have been described which could have indirectly affected ALP metabolism in the Nursehound Shark (46). Another factor to consider in the evaluation of ALP variations is the effect of capture-related stress, as ALP levels have been associated with tissue damage in severely stressed fish during long and intense capture procedures (>50 min), showing a positive relationship with capture time, although the differences observed in our study are less probably related to this as all procedures were consistently short (41). Median ALP values reported in this study (7.7 U/l) were kept within the general ALP range described for elasmobranchs and previously reported values for other elasmobranch species such as the free-ranging Bull Shark (*Carcharhinus leucas*) (median 2 U/l), the free-ranging Bonnethead Shark (median 5 U/l), the free-ranging Grey Nurse Shark (median 20 U/l), and the aquarium-maintained Cownose Ray (median 34 U/l) (12, 34, 42). Variations in diet components, environmental parameters, capture time and age can also lead to alterations in calcium and phosphorus, though no significant differences in these analytes were detected between aquaria in this study and median values (3.97 and 1.58 mmol/l, respectively) were kept within values previously reported in wild-caught Cownose Rays, Southern Stingrays, Bonnethead Shark, and the aquarium-maintained Smooth Dogfish (8, 16, 30, 36, 43).

Median GGT levels were significantly higher in shark classified as adults (0.7 U/l) compared to subadults (0.4 U/l) (*p* < 0.05; Mann-Whitney *U* test). This enzyme, which is present in cell membranes, contributes to maintain intracellular homeostasis and is a physiological biomarker of hepatobiliary disfunction, increasing in sharks with bile accumulation (27, 44). Variations of GGT with increasing age have been described not only in mammals, but also in other shark species such as the free-ranging Grey Nurse Shark, with lower GGT values in juvenile (0–4 U/l) compared to adult individuals (1–8 U/l) (27, 47). Despite being under the linear range of detection (3–1,200 U/l), RI determined for GGT activity in this study (0.0–2.6 U/l) were similar to those previously determined in other shark species such as the free-ranging Blue Shark (*Prionacea glauca*) (0–2 U/l) and the free-ranging Scalloped Hammerhead (*Sphyrna lewini*) (0–4 U/l) (27).

Creatine kinase values determined in this study were below the instrument range (<10 U/l). Reported plasma CK values vary greatly between the different studies involving elasmobranchs; median CK was 80.5 U/l in wild-caught southern stingrays, values ranged from 18 to 735 U/l in wild-caught Bonnethead Sharks, low levels (2–13 U/l) were described in Smooth Dogfish under human care, and CK levels under the limit of detection (<4 U/l) were also reported in aquarium-maintained Cownose Rays (8, 16, 30, 34). Important differences between studies and elasmobranch species have also been de-scribed for AST; Nursehound Shark median AST values (7.6 U/l) were under those reported in wild-caught Atlantic Sharpnose shark (*Rhizoprionodon terraenovae*) (22.5 U/l) and Bonnethead Shark (39.0 U/l), though very close to those of wild-caught Spiny Dogfish (7.0 U/l) and aquarium maintained Smooth Dogfish (10.6 U/l) (15, 30). In this study, LDH values were below the instrument range (<25 U/l). This is consistent with the low LDH levels, frequently under the limit of detection, reported in other elasmobranch species such as wild-caught Bonnethead Shark (<5 U/l), aquarium-maintained Smooth Dogfish (<10 U/l), and Cownose Ray (<4 U/l) (16, 30, 34). Previous studies have shown that CK, AST, and LDH enzymes, normally found intracellularly, significantly increase in plasma after the capture and transport of elasmobranchs, indicating cell disruption and potential necrosis, and suggesting the presence of muscle damage in elasmobranchs (48). The mentioned differences highlight the need to carefully describe capture procedures and sampling technique when establishing blood RV in elasmobranchs to avoid capture-related stress effects on analytical data (12). Former studies already demonstrated the importance of using well-defined standard procedures when establishing RI in elasmobranchs, including restraint and sampling methods which are fast and efficient, to reduce capture-related stress artifacts on analytical data (12, 49). Due to the short sampling procedure required for sample collection during this study, and as anesthesia and chemical sedation have shown to affect stress-related blood variables in sharks, tonic immobility was selected in this study as the restraint method of choice (50). Tonic immobility is described as an effective immobilization method in sharks for non-painful, short handling procedures, reducing the chances of trauma and avoiding the complications associated with chemical immobilization (22). Despite tonic immobility has also been described to produce physiological changes in elasmobranchs when maintained in this state for extended periods of time, the quick sampling procedure developed in this study is considered the least invasive and has been used for blood collection in different physiological, hematological and plasma chemical studies with elasmobranchs, including sharks from the family *Scyliorhinidae* (12, 14, 22, 50, 51). As previously described in other catshark species, placing Nursehound Shark in a dorsoventral decubitus rapidly induced a state of tonic immobility (<1 min), which allowed the safe and efficient handling of the animals; the mild sedation produced during tonic immobility was rapidly reversed (<1 min) when shark were turned ventrodorsally after sampling (22). In the design of this study, blood collection was performed prior to morphological measurement to decrease time to blood collection and therefore capture-related effects on blood analytics (49). The same capture and animal handling protocol was used for all shark included in this study, and blood collection and sample processing was performed by the same researcher and sample equipment for all animals in an attempt to reduce possible variations in the obtained analytical values. Blood RV provided in this studycould serve as a useful tool for researchers or members of animal care and welfare committees evaluating the effect of capture, transport, handling, and different experimental procedures on the analytical values of this species.

Slight non-statistically significant variations in glucose values were detected across aquaria as median levels were higher in Oceanogràfic Aquarium (0.91 mmol/l) than in Finisterrae Aquarium (0.83 mmol/l), Bioparc Gijón Aquarium (0.80 mmol/l), Museo Marítimo del Cantábrico (0.85 mmol/l), and Sevilla Aquarium (0.86 mmol/l). Previous studies have determined that glucose can decrease by 5–7% per hour in unprocessed blood samples due to glycolysis (52). Variations in processing time (always under 90 min) could have contributed to the variations in glucose values obtained in this study and should be considered when determining glucose RV in elasmobranchs. Previous studies reported low glucose values as a normal finding in healthy elasmobranchs and obtained similar values to those provided in this study (14). Other factors including the influence of hyperactivity and stress associated with capture and handling should also be considered when determining glucose values, though interpretation should be performed with caution in elasmobranchs due to the important physiological and metabolic differences compared to other aquatic vertebrates (14, 53, 54). Further studies evaluating the effect of handling and tonic immobility on plasma chemical values are needed in the Nursehound Shark to allow an accurate interpretation of variations produced in this analyte.

Electrolyte values (calcium, chloride, sodium, and potassium) obtained in this study were similar to those previously reported in other elasmobranchs maintained under human care such as the Smooth Dogfish, the Cownose Ray, and the Southern Stingray (14, 30, 34). No statistically significant differences in electrolyte values were detected in this study between sexes nor developmental stages. Median BUN value reported in this study (281.6 mmol/l) were lower than those for Spiny Dogfish (350.35 mmol/l) and cownose rays under human care (363.10 mmol/l) (14, 30). Variations in BUN within different elasmobranch species have been previously described, with BUN values normally high in healthy elasmobranchs (8). In addition, elasmobranch BUN is directly influenced by nutritional status, as these animals can transform ammonia into urea (21, 55). To avoid imbalances in analyte measurements, individualswere fasted for a minimum 24 h period; this could have influenced lower BUN values, as previous studies using Spiny Dogfish showed a significant increase in BUN 20 h after feeding (21). Furthermore, BUN values were closer to those reported in wild-caught Cownose Ray following transport (304.96 mmol/l), an stressful procedure in which animals normally do not eat (36). It should be also considered that previous physiological studies with elasmobranchs reported a significant increase in plasma electrolytes, together with a decrease in BUN values after a stressful stimulus such as an 18 min period of aerial exposure (50). In the mentioned study, urea did not decrease significantly right after the aerial exposure, though it was significantly reduced 5 h after the stressful procedure. Urea is the main organic osmolyte in marine elasmobranchs, and the decreased BUN values in marine elasmobranchs has been linked to the increased permeability of the gills produced during the acute-stress response, which improves gas exchange and allows the passive diffusion of ions (56). This mechanism leads to an increase in plasma osmolarity, with increased levels of calcium, chloride, sodium and potassium in plasma, together with a decrease in BUN, which is mainly eliminated in the gills in exchange with sodium (50, 56). These variations also illuminate the importance of registering and considering environmental conditions, diet, and sampling procedures and time to blood collection used in studies measuring blood RV in elasmobranchs. In this study, although slight variations were observed in calcium, chloride, sodium, potassium, and BUN between aquaria, differences were not statistically significant (*p* > 0.05; Kruskal-Wallis, Bonferroni-adjusted), likely due to the similar environmental and management conditions and equal sampling protocols.

Plasma total cholesterol values were significantly higher in males (median 2.17 mmol/l) when compared to females (median 1.71 mmol/l) (*p* < 0.01). Important variations in cholesterol and lipoproteins have been described according to species, season, and reproductive status in elasmobranchs, also showing higher cholesterol levels in male Small-spotted Catshark when compared to females (12, 57). Statistical comparison between animals classified as adult females vs. adult males also showed significant differences (*p* < 0.05; Dunn's test, Bonferroni-adjusted), but the difference between subadult males and subadult females was not significant (*p* = 0.295; Dunn's test, Bonferroni-adjusted). Similar to cholesterol, variations in plasma triglyceride levels have been reported in elasmobranchs related to sexual maturation, reproduction, and food availability (58). Although former studies with free-ranging Small-spotted Catshark described significantly higher plasma triglycerides in females compared to male shark associated with vitellogenesis, no significant differences in triglyceride levels were detected in our study between sexes (57). Despite reproductive behavior including copulation was observed between adults, and females were laying fertile eggs throughout the year in all aquaria (except for Bioparc Gijón Aquarium), shark included in the study were not individually identified and thus it was not possible to determine which females were carrying eggs. Therefore, we were unable to evaluate differences between inactive and cycling females. Nursehound Shark had similar plasma triglyceride (median 0.97 mmol/l) and total cholesterol (2.05 mmol/l) levels compared to those previously reported in their close relative the Small-spotted Catshark (1.27 mmol/l and 2.85 mmol/l, respectively) and in other elasmobranchs under human care such as the Spotted Eagle Ray (0.89 mmol/l and 2.56 mmol/l, respectively) and were higher than those reported in free-ranging Grey Nurse Shark (0.30 mmol/l and 1.40 mmol/l, respectively) (12, 14, 31, 34, 58). The lowest triglyceride and cholesterol values observed in free-ranging elasmobranchs compared to those under human care could indicate a lower energy deficit in animals maintained un aquaria, which are regularly fed, suggesting that triglyceride levels could turn into a useful indicator for health assessments (14, 36, 59).

In our study, no significant differences were detected between animals sampled from the different aquaria for total cholesterol or triglycerides (*p* > 0.05; Dunn's test, Bonferroni-adjusted). Correlation coefficient between weight and triglyceride levels, as well as between triglyceride levels and total cholesterol levels was very low (R^2^ = 0.004 and 0.005, respectively). Caution should be taken when interpreting cholesterol levels in fish, as this molecule is a precursor of steroidal hormones, an increased values have been reported after stressful stimuli such as aerial exposure or anesthesia for procedures over 5 min (60). The absence of significant variations in total cholesterol and triglyceride levels across the sampled aquaria would support the absence of significant differences in body condition within the studied individuals, as well as suggest that the sampling protocol developed in this study was consistent, reducing capture-related stress and its effects on analyte values. In this study, we were unable to evaluate possible differences in hematology and plasma chemistry linked to seasonal variations, as all animals were sampled during the same season (summer). Further studies sampling Nursehound Shark in different seasons and using individually identified animals, will allow for the classification of females according to their reproductive stage, and will help to better understand some of these variations in analytical values related to gestation in this species.

## Conclusions

Hematology and plasma chemistry data provided in this study can be used as a valuable health assessment tool for nursehounds under human care. These results will contribute to the limited baseline data available for professionals working with catsharks and serve to increase our understanding of blood analytics for future *in situ* and *ex situ* studies of elasmobranchs.

## Data Availability Statement

The raw data supporting the conclusions of this article will be made available by the authors, without undue reservation.

## Ethics Statement

The animal study was reviewed and approved by Animal Care and Welfare Committee at Oceanogràfic of Valencia and the Generalitat of Valencia (project reference ID OCE-22-19).

## Author Contributions

Study design: PM-E and DG-P. Data acquisition: PM-E, CR-S, TA-A, and MV-T. Data processing: PM-E and CS. Preparation of the manuscript: PM-E. Revision of the manuscript: DG-P, CR-S, and TE. All authors have read and agreed to the published version of the manuscript.

## Funding

Funding for this study was provided by the Complutense University of Madrid Predoctoral Contract (PME; CT63/19-CT64/19) and the Fundación Oceanogràfic (Under the project reference number OCE-22-19).

## Conflict of Interest

The authors declare that the research was conducted in the absence of any commercial or financial relationships that could be construed as a potential conflict of interest.

## Publisher's Note

All claims expressed in this article are solely those of the authors and do not necessarily represent those of their affiliated organizations, or those of the publisher, the editors and the reviewers. Any product that may be evaluated in this article, or claim that may be made by its manufacturer, is not guaranteed or endorsed by the publisher.
