# Supplementary material for: Hematology and Plasma Chemistry Reference Values in Nursehound Shark (Scyliorhinus Stellaris) Maintained Under Human Care
Source: Front Vet Sci. 2022 Jul 11;9:909834. doi: 10.3389/fvets.2022.909834 (PMC9310093; doi:10.3389/fvets.2022.909834)
Supplement: Supplementary file 1 [file Data_Sheet_1.docx]

Supplementary Material

# Supplementary Tables

**Supplementary Table 1**. Morphometrical measurements (female *vs* male) Nursehound Shark (*Scyliorhinus stellaris*) maintained under human care. Sex was determined based on the presence of claspers. No statistically significant differences were detected between sexes (*p* > 0.05; Mann-Whitney U test).

|  | **Female** | | | | | | **Male** | | | | | |  |
| --- | --- | --- | --- | --- | --- | --- | --- | --- | --- | --- | --- | --- | --- |
| **Measurement (unit)** | **n** | **Mean** | **Median** | **SD** | **Min** | **Max** | **n** | **Mean** | **Median** | **SD** | **Min** | **Max** | **P** |
| Weight (kg) | 47 | 2.7 | 2.4 | 1.1 | 0.8 | 5.0 | 47 | 2.8 | 2.4 | 1.6 | 0.7 | 7.5 | 0.817 |
| TL (cm) | 47 | 80.3 | 78.0 | 12.8 | 53.0 | 103.0 | 47 | 81.3 | 80.0 | 15.2 | 51.0 | 113.0 | 0.607 |
| SVL (cm) | 47 | 38.9 | 38.0 | 6.9 | 26.0 | 53.0 | 47 | 39.7 | 39.0 | 8.4 | 18.0 | 56.0 | 0.522 |

n, number of individuals; SD, standard deviation; P, *p*-value; TL, total length (measured from the tip of the snout to the tip of the longer lobe of the caudal fin); SVL, snout to vent length (measured from the tip of the snout to cloacal opening).

**Supplementary Table 2.** Main hematology values (female *vs* male) determined in Nursehound Sharks (*Scyliorhinus stellaris*) under human care. No statistically significant differences were detected between sexes (*p* > 0.05; Mann-Whitney U test).

|  |  | | **Female** | | | | |  | | **Male** | | | |  |
| --- | --- | --- | --- | --- | --- | --- | --- | --- | --- | --- | --- | --- | --- | --- |
| **Analyte (unit)** | **n** | **Mean** | | **SD** | **Median** | **Min** | **Max** | **n** | **Mean** | **SD** | **Median** | **Min** | **Max** | **P** |
| PCV (l/l) | 47 | 0.17 | | 0.02 | 0.18 | 0.14 | 0.21 | 47 | 0.17 | 0.10 | 0.17 | 0.15 | 0.20 | 0.259 |
| TS (g/l) | 47 | 58.0 | | 5.0 | 58.0 | 50.0 | 66.0 | 47 | 58.0 | 5.0 | 58.0 | 50.0 | 68.0 | 0.560 |
| WBC (10^9^/l) | 42 | 8.86 | | 2.22 | 8.40 | 5.91 | 14.53 | 44 | 8.85 | 2.83 | 8.22 | 4.91 | 14.25 | 0.327 |
| RBC (10^9^/l) | 47 | 169.9 | | 20.4 | 172.5 | 132.5 | 210.0 | 47 | 175.6 | 24.3 | 180.0 | 122.5 | 240.0 | 0.190 |
| L (%) | 42 | 39.0 | | 5.1 | 38.0 | 29.0 | 54.0 | 44 | 39.9 | 5.9 | 40.0 | 27.0 | 51.0 | 0.520 |
| M (%) | 42 | 3.1 | | 1.6 | 3.0 | 0.0 | 7.0 | 44 | 2.6 | 1.6 | 2.0 | 0.0 | 6.0 | 0.227 |
| N (%) | 42 | 2.4 | | 1.6 | 2.0 | 0.0 | 6.0 | 44 | 2.5 | 1.6 | 2.0 | 0.0 | 6.0 | 0.764 |
| FEG (%) | 42 | 6.4 | | 2.7 | 6.0 | 2.0 | 13.0 | 44 | 6.2 | 2.9 | 6.0 | 1.0 | 14.0 | 0.468 |
| CEG (%) | 42 | 20.5 | | 5.2 | 19.0 | 12.0 | 33.0 | 44 | 20.9 | 4.1 | 21.5 | 12.0 | 30.0 | 0.314 |
| GT (%) | 42 | 28.5 | | 6.5 | 29.5 | 12.0 | 40.0 | 44 | 27.9 | 6.3 | 28.0 | 12.0 | 40.0 | 0.988 |
| L (10^9^/l) | 41 | 3.39 | | 0.94 | 3.25 | 1.79 | 5.74 | 43 | 3.49 | 1.32 | 3.24 | 1.55 | 6.55 | 0.513 |
| M (10^9^/l) | 42 | 0.27 | | 0.16 | 0.23 | 0.00 | 0.65 | 44 | 0.22 | 0.13 | 0.22 | 0.00 | 0.57 | 0.677 |
| N (10^9^/l) | 41 | 0.20 | | 0.13 | 0.18 | 0.00 | 0.52 | 43 | 0.21 | 0.14 | 0.19 | 0.00 | 0.56 | 0.400 |
| FEG (10^9^/l) | 42 | 0.56 | | 0.27 | 0.47 | 0.22 | 1.32 | 44 | 0.53 | 0.28 | 0.49 | 0.09 | 1.29 | 0.529 |
| CEG (10^9^/l) | 42 | 1.80 | | 0.59 | 1.73 | 0.92 | 3.63 | 44 | 1.85 | 0.68 | 1.66 | 0.69 | 3.67 | 0.862 |
| GT (10^9^/l) | 42 | 2.52 | | 0.80 | 2.46 | 0.75 | 4.41 | 43 | 2.39 | 0.90 | 2.10 | 1.13 | 4.50 | 0.513 |

n, number of individuals; SD, standard deviation; P, *p*-value; PCV, packed cell volume; TS, total solids; WBC, white blood cells; RBC, red blood cells; L, lymphocyte; M, monocyte; FEG, fine eosinophilic granulocyte; CEG, coarse eosinophilic granulocyte; N, neutrophile; GT, granulated thrombocyte.

**Supplementary Table 3.** Main hematology values (adult *vs* subadult) determined in Nursehound Sharks (*Scyliorhinus stellaris*) under human care. No statistically significant differences were detected between developmental stages (*p* > 0.05; Mann-Whitney U test).

|  | **Adult** | | | | | | **Subadult** | | | | | |  |
| --- | --- | --- | --- | --- | --- | --- | --- | --- | --- | --- | --- | --- | --- |
| **Analyte (unit)** | **n** | **Mean** | **SD** | **Median** | **Min** | **Max** | **n** | **Mean** | **SD** | **Median** | **Min** | **Max** | **P** |
| PCV (l/l) | 54 | 0.17 | 0.02 | 0.17 | 0.14 | 0.21 | 40 | 0.17 | 0.01 | 0.17 | 0.15 | 0.20 | 0.815 |
| TS (g/l) | 54 | 58.3 | 4.3 | 59.5 | 50.0 | 68.0 | 40 | 57.1 | 4.7 | 56.0 | 50.0 | 66.0 | 0.174 |
| WBC (10^9^/l) | 47 | 8.95 | 2.69 | 8.61 | 4.91 | 14.53 | 39 | 8.73 | 2.36 | 8.10 | 5.53 | 13.68 | 0.818 |
| RBC (10^9^/l) | 54 | 171.5 | 26.5 | 176.2 | 122.5 | 240.0 | 40 | 1744.4 | 15.6 | 177.5 | 142.5 | 205.0 | 0.579 |
| L (%) | 47 | 39.8 | 5.7 | 40.0 | 27.0 | 54.0 | 39 | 39.1 | 5.2 | 38.0 | 28.0 | 54.0 | 0.485 |
| M (%) | 47 | 2.9 | 1.6 | 3.0 | 0.0 | 7.0 | 39 | 2.8 | 1.7 | 2.0 | 0.0 | 6.0 | 0.629 |
| N (%) | 47 | 2.5 | 1.7 | 2.0 | 0.0 | 6.0 | 39 | 2.5 | 1.4 | 2.0 | 0.0 | 6.0 | 0.435 |
| FEG (%) | 47 | 6.5 | 2.9 | 6.0 | 1.0 | 14.0 | 39 | 6.1 | 2.6 | 6.0 | 1.0 | 12.0 | 0.776 |
| CEG (%) | 47 | 22.1 | 4.7 | 22.0 | 13.0 | 33.0 | 39 | 20.3 | 4.2 | 20.0 | 14.0 | 30.0 | 0.358 |
| GT (%) | 47 | 26.6 | 6.7 | 28.0 | 12.0 | 40.0 | 39 | 29.2 | 5.7 | 30.0 | 14.0 | 38.0 | 0.183 |
| L (10^9^/l) | 45 | 3.44 | 1.20 | 3.11 | 1.78 | 6.55 | 39 | 3.44 | 1.10 | 3.36 | 1.55 | 6.02 | 0.669 |
| M (10^9^/l) | 47 | 0.25 | 0.15 | 0.23 | 0.00 | 0.62 | 39 | 0.24 | 0.15 | 0.20 | 0.00 | 0.65 | 0.834 |
| N (10^9^/l) | 45 | 0.19 | 0.14 | 0.17 | 0.00 | 0.56 | 39 | 0.22 | 0.13 | 0.19 | 0.00 | 0.52 | 0.275 |
| FEG (10^9^/l) | 47 | 0.56 | 0.28 | 0.52 | 0.12 | 1.21 | 39 | 0.53 | 0.27 | 0.46 | 0.09 | 1.32 | 0.746 |
| CEG (10^9^/l) | 47 | 1.89 | 0.73 | 1.84 | 0.69 | 3.67 | 39 | 1.74 | 0.50 | 1.65 | 1.09 | 3.63 | 0.497 |
| GT (10^9^/l) | 47 | 2.42 | 0.83 | 2.45 | 0.75 | 4.00 | 38 | 2.50 | 0.87 | 2.29 | 1.13 | 4.50 | 0.712 |
|  |  |  |  |  |  |  |  |  |  |  |  |  |  |

n, number of individuals; SD, standard deviation; P, *p*-value; PCV, packed cell volume; TS, total solids; WBC, white blood cells; RBC, red blood cells; L, lymphocyte; M, monocyte; FEG, fine eosinophilic granulocyte; CEG, coarse eosinophilic granulocyte; N, neutrophil; GT, granulated thrombocyte.

**Supplementary Table 4.** Main blood chemistry values (female *vs* male) determined in Nursehound Sharks (*Scyliorhinus stellaris*) under human care. Bold font and superscripts indicate statistically significant differences between sexes (*p* < 0.05; Mann-Whitney U test).

|  | **Female** | | | | | | **Male** | | | | | |  |
| --- | --- | --- | --- | --- | --- | --- | --- | --- | --- | --- | --- | --- | --- |
| **Analyte (unit)** | **n** | **Mean** | **SD** | **Median** | **Min** | **Max** | **n** | **Mean** | **SD** | **Median** | **Min** | **Max** | **P** |
| ALP (U/l) | 46 | 8.0 | 2.2 | 7.8 | 4.1 | 13.4 | 46 | 7.5 | 2.2 | 7.3 | 4.1 | 13.8 | 0.327 |
| AST (U/l) | 45 | 8.1 | 3.6 | 7.5 | 3.3 | 17.2 | 43 | 7.9 | 3.5 | 7.6 | 3.0 | 16.9 | 0.894 |
| BUN (mmol/l) | 47 | 282.9 | 12.9 | 284.2 | 256.0 | 306.7 | 47 | 280.9 | 12.0 | 280.3 | 262.8 | 315.7 | 0.354 |
| Ca (mmol/l) | 45 | 3.94 | 0.22 | 3.94 | 3.59 | 4.62 | 44 | 4.02 | 0.20 | 4.02 | 3.52 | 4.37 | 0.072 |
| **Chol (mmol/l)** | 47 | **1.79** | 0.56 | **1.71^a^** | 0.60 | 3.21 | 46 | **2.36** | 0.80 | **2.17 ^b^** | 1.13 | 4.04 | **< 0.05** |
| Cl (mmol/l) | 43 | 232.6 | 9.1 | 232.0 | 216.0 | 259.0 | 43 | 234.3 | 10.9 | 235.0 | 213.0 | 259.0 | 0.387 |
| Fe (µmol/l) | 47 | 3.79 | 1.32 | 3.87 | 1.27 | 7.00 | 47 | 3.78 | 1.38 | 3.79 | 1.75 | 6.95 | 0.910 |
| GGT (U/l) | 47 | 1.0 | 0.9 | 0.6 | 0.0 | 2.6 | 44 | 0.8 | 0.7 | 0.4 | 0.1 | 2.8 | 0.351 |
| Gluc (mmol/l) | 45 | 0.88 | 0.20 | 0.86 | 0.52 | 1.36 | 45 | 0.89 | 0.26 | 0.87 | 0.44 | 1.44 | 0.747 |
| K (mmol/l) | 47 | 3.8 | 0.4 | 3.8 | 2.8 | 4.4 | 47 | 3.8 | 0.4 | 3.7 | 3.0 | 4.8 | 0.639 |
| Na (mmol/l) | 47 | 244.0 | 11.2 | 241.0 | 224.0 | 271.0 | 45 | 245.5 | 11.1 | 244.0 | 227.0 | 272.0 | 0.300 |
| Phos (mmol/l) | 47 | 1.55 | 0.29 | 1.55 | 1.00 | 2.13 | 47 | 1.61 | 0.26 | 1.61 | 1.03 | 2.13 | 0.390 |
| TP (g/l) | 47 | 25.0 | 4.0 | 24.0 | 20.0 | 36.0 | 47 | 25.0 | 4.0 | 24.0 | 19.0 | 35.0 | 0.817 |
| Trig (mmol/l) | 44 | 1.25 | 0.71 | 0.97 | 0.39 | 3.44 | 46 | 1.09 | 0.58 | 0.95 | 0.48 | 3.37 | 0.240 |

n, number of individuals; SD, standard deviation; RI, reference intervals; LRL, 90% confidence interval of the lower reference limit; URL, 90% confidence interval of the upper reference limit; P, *p*-value; ALP, alkaline phosphatase; AST, aspartate aminotransferase; BUN, blood urea nitrogen; Ca, calcium; Chol, total cholesterol; Cl, chloride; GGT, gamma-glutamyl transferase; Gluc, glucose; Fe, iron; Na, sodium; Phos, phosphorus; K, potassium; TP, total proteins; Trig, triglycerides. Reported GGT values were under the linear range of detection of the chemistry analyzer (3-1200 U/l).

**Supplementary Table 5.** Main blood chemistry values (adult *vs* subadult) determined in Nursehound Sharks (*Scyliorhinus stellaris*) under human care. Bold font and superscripts indicate statistically significant differences between developmental stages (*p* < 0.05; Mann-Whitney U test).

|  |  | | **Adult** | | | | | |  | | **Subadult** | | | | | |  |
| --- | --- | --- | --- | --- | --- | --- | --- | --- | --- | --- | --- | --- | --- | --- | --- | --- | --- |
| **Analyte (unit)** | **n** | **Mean** | |  | **SD** | **Median** | **Min** | **Max** | **n** | **Mean** | |  | **SD** | **Median** | **Min** | **Max** | **P** |
| ALP (U/l) | 53 | 7.5 | |  | 2.4 | 7.1 | 4.1 | 13.8 | 39 | 8.1 | |  | 1.8 | 7.9 | 4.5 | 12.0 | 0.134 |
| AST (U/l) | 49 | 8.3 | |  | 3.7 | 8.0 | 3.0 | 17.2 | 39 | 7.7 | |  | 3.3 | 7.2 | 3.3 | 16.9 | 0.340 |
| BUN (mmol/l) | 54 | 282.7 | |  | 13.6 | 284.3 | 260.0 | 315.7 | 40 | 280.6 | |  | 10.7 | 279.9 | 265.7 | 305.6 | 0.535 |
| Ca (mmol/l) | 51 | 3.99 | |  | 0.25 | 3.94 | 3.52 | 4.62 | 38 | 3.97 | |  | 0.15 | 3.99 | 3.67 | 4.37 | 0.733 |
| Chol (mmol/l) | 53 | 2.01 | |  | 0.82 | 1.81 | 0.60 | 4.04 | 40 | 2.16 | |  | 0.62 | 2.06 | 1.26 | 3.45 | 0.131 |
| Cl (mmol/l) | 48 | 231.6 | |  | 11.1 | 229.0 | 213.0 | 259.0 | 38 | 235.8 | |  | 7.9 | 235.0 | 216.0 | 259.0 | 0.067 |
| Fe (µmol/l) | 54 | 3.92 | |  | 1.36 | 3.90 | 1.75 | 7.00 | 40 | 3.60 | |  | 1.31 | 3.42 | 1.27 | 6.89 | 0.236 |
| **GGT (U/l)** | 51 | **1.1** | |  | 0.9 | **0.7 ^a^** | 0.0 | 2.8 | 40 | **0.6** | |  | 0.6 | **0.4 ^b^** | 0.0 | 2.6 | **< 0.05** |
| Gluc (mmol/l) | 50 | 0.92 | |  | 0.24 | 0.89 | 0.44 | 1.44 | 40 | 0.84 | |  | 0.21 | 0.84 | 0.47 | 1.36 | 0.068 |
| K (mmol/l) | 54 | 3.8 | |  | 0.4 | 3.8 | 2.9 | 4.8 | 40 | 3.8 | |  | 0.4 | 3.8 | 2.8 | 4.4 | 0.957 |
| Na (mmol/l) | 52 | 244.8 | |  | 12.6 | 241.0 | 227.0 | 272.0 | 40 | 244.7 | |  | 9.0 | 244.0 | 224.0 | 271.0 | 0.275 |
| Phos (mmol/l) | 54 | 1.58 | |  | 0.29 | 1.61 | 1.00 | 2.13 | 40 | 1.58 | |  | 0.22 | 1.58 | 1.13 | 2.03 | 0.863 |
| TP (g/l) | 54 | 26.0 | |  | 4.0 | 26.0 | 1.9 | 36.0 | 40 | 24.0 | |  | 4.0 | 23.0 | 2.0 | 33.0 | 0.0598 |
| Trig (mmol/l) | 50 | 1.17 | |  | 0.64 | 1.03 | 0.52 | 3.44 | 40 | 1.16 | |  | 0.66 | 0.87 | 0.38 | 3.37 | 0.826 |

n, number of individuals; SD, standard deviation; RI, reference intervals; LRL, 90% confidence interval of the lower reference limit; URL, 90% confidence interval of the upper reference limit; P, *p*-value; ALP, alkaline phosphatase; AST, aspartate aminotransferase; BUN, blood urea nitrogen; Ca, calcium; Chol, total cholesterol; Cl, chloride; GGT, gamma-glutamyl transferase; Gluc, glucose; Fe, iron;; Na, sodium; Phos, phosphorus; K, potassium; TP, total proteins; Trig, triglycerides. Reported GGT values were under the linear range of detection of the chemistry analyzer (3-1200 U/l).
